# Inverted (p–i–n) perovskite solar cells using a low temperature processed TiO_*x*_ interlayer†
†Electronic supplementary information (ESI) available: Detail synthesis of NiO_*x*_ nanoparticles, organic salts, and TiO_*x*_ sol–gel are included. See DOI: 10.1039/c8ra03993c


**DOI:** 10.1039/c8ra03993c

**Published:** 2018-07-10

**Authors:** Bekele Hailegnaw, Getachew Adam, Herwig Heilbrunner, Dogukan H. Apaydin, Christoph Ulbricht, Niyazi Serdar Sariciftci, Markus C. Scharber

**Affiliations:** a Linz Institute for Organic Solar Cells (LIOS), Institute of Physical Chemistry, Johannes Kepler University Linz, Altenbergerstrasse 69, 4040 Linz, Austria. Email: markus_clark.scharber@jku.at; b Department of Industrial Chemistry, College of Applied Science, Addis Ababa Science and Technology University (AASTU), P. O. Box 16417, Addis Ababa, Ethiopia; c Institute of Polymer Materials and Testing (IPMT), Johannes Kepler University Linz, Altenbergerstrasse 69, 4040 Linz, Austria

## Abstract

In this article, we present the improvement in device performance and stability as well as reduction in hysteresis of inverted mixed-cation–mixed-halide perovskite solar cells (PSCs) using a low temperature, solution processed titanium oxide (TiO_*x*_) interlayer between [6,6]-phenyl-C_61_ butyric acid methyl ester (PCBM) and an Al electrode. Upon applying a TiO_*x*_ interlayer, device resistance was reduced compared to that of the control devices, which results in improved rectification of the characteristic current density–voltage (*J*–*V*) curve and improved overall performance of the device. PSCs with the TiO_*x*_ interlayer show an open-circuit voltage (*V*_oc_) of around 1.1 V, current density (*J*_sc_) of around 21 mA cm^–2^, fill factor (FF) of around 72% and enhanced power conversion efficiency (PCE) of 16% under AM1.5 solar spectrum. Moreover, devices with the TiO_*x*_ interlayer show improved stability compared to devices without the TiO_*x*_ interlayer. This finding reveals the dual role of the TiO_*x*_ interlayer in improving device performance and stability.

## Abstract 2

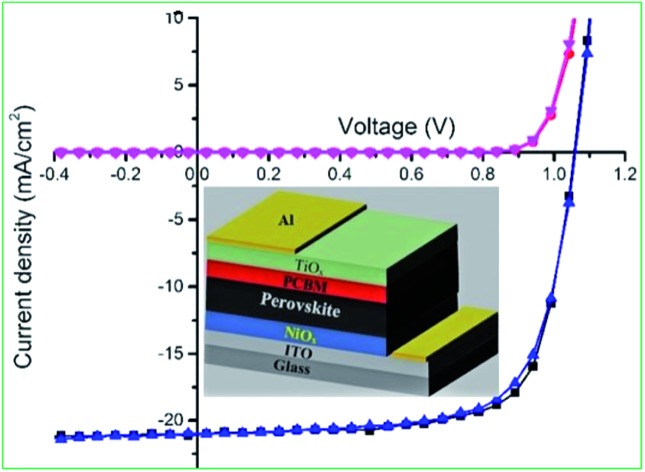
A low temperature solution processed TiO_*x*_ interlayer was used to improve the performance and stability of mixed-cation–mixed-halide perovskite solar cells.

## Introduction

Solar cells based on hybrid organic–inorganic (metal halide) perovskite materials are a dominant research topic, which has caught the interest of many researchers in the area of photovoltaics. Since Miyasaka *et al.* (2009) reported the first hybrid organic–inorganic perovskite solar cell with power conversion efficiency (PCE) of 3.8%, astonishing progress has been made. By 2012, the PCE of such hybrid organic–inorganic perovskite solar cells was increased to 9.7% by replacing liquid electrolyte with a solid organic hole transporting material (spiro-OMeTAD).^1^–^3^ This motivated many researchers to further develop these materials. Since then, various fabrication methods have been applied and many exciting theoretical and experimental studies have been performed to understand the optoelectrical properties of these semiconductors.^4^–^10^ Currently, the record efficiency exceeds 22.7% in small-area cells and 16% in large-area modules (above 1 cm^2^ area) for n–i–p (also called regular PSCs) configured perovskite solar cells (PSCs).^11^–^13^ For inverted (p–i–n) type PSCs, the PCE exceeds 19%.^14^–^17^ Apart from the exceptional PCE improvement, hybrid organic–inorganic perovskite materials possess several appealing properties, particularly easy solution processability, high absorption coefficients, low exciton binding energy, long and balanced carrier diffusion paths (a property of high mobility and long charge lifetimes), high structural defect tolerance, shallow intrinsic defects and benign grain boundary effects. Also, the bandgap can be tuned to a large extent by choice of metal cation, inorganic anion, and organic cation.^18^–^21^

To improve performance of PSCs, a wide range of advanced structural^22^–^24^ and compositional^10^,^12^,^13^,^16^,^17^,^25^ engineering options have been investigated. It has been shown that interfacial engineering plays a significant role in improving carrier extraction and overall performance of PSCs.^26^–^29^ Nevertheless, the issues of stability and device hysteresis remain challenging for developing PSCs.^30^–^34^

One of the promising structural engineering innovations in inverted or p–i–n type PSCs is the introduction of electron transporting layers (ETLs), which are mostly based on [6,6]-phenyl-C_61_ butyric acid methyl ester (PCBM). Also, other non-fullerene organic and inorganic acceptors have been used as ETLs.^26^,^35^–^38^ However, an injection barrier has been observed at the interface of PCBM and metal electrodes (Al, Ag and/or Au).^26^,^39^,^40^ The barrier height formed between the Fermi level of the metal electrode and the highest occupied molecular orbital (LUMO) level of PCBM causes charge carrier extraction resistance and hence reduces electron extraction and overall performance of the cell.^26^,^39^,^41^ Reducing the effective work function of a metal electrode and tuning the energy level alignment with the ETL (n-type semiconductor) can be achieved by incorporating interfacial dipole layers such as LiF, TiO_*x*_, poly(2-ethyl-2-oxazoline) (PEOz), ZnO, MgF_2_, MgO, and 2,9-dimethyl-4,7-diphenyl-1,10-phenanthroline (BCP).^26^,^42^–^45^ Docampo *et al.*^38^ demonstrated the possibility of using TiO_*x*_ as an interfacing layer in inverted mixed halide based PSCs.

In this study, we prepared inverted mixed-cation–mixed-halide PSCs based on nickel oxide (NiO_*x*_) and poly(3,4-ethylenedioxythiophene)–poly(styrenesulfonate) (PEDOT:PSS) hole transporting layers (HTLs). We investigated the effect of a layer of colloidal TiO_*x*_ particles, which was processed from solution at low temperatures and deposited between PCBM and the aluminum back contact. We found a reduction in the serial resistance, an increase in the recombination resistance across the interface, an improvement in the overall performance of PSCs and better stability when TiO_*x*_ was applied as the interfacial layer between PCBM and Al electrode.

## Experimental section

### a. Materials

We used patterned indium-doped tin oxide (ITO) coated glass substrates (15 Ω cm^–2^), lead iodide (PbI_2_, Sigma Aldrich, 99.9%), lead bromide (PbBr_2_, Sigma Aldrich, 99.99%), [6,6]-phenyl-C_61_-butyric acid methyl ester (PCBM, Solenne BV), PEDOT:PSS (Clevios F HC Solar, SCA 418-12), nickel chloride hexahydrate (NiCl_2_·6H_2_O, Sigma Aldrich, 99.9%), cesium iodide (CsI, Sigma Aldrich, 99.99%), and sodium hydroxide (NaOH, Sigma Aldrich, ≥98%). Methyl ammonium bromide (MABr), methyl ammonium iodide (MAI) and formamidinium iodide (FAI) were synthesized in our lab, as mentioned in the synthesis of organic halides. Titanium(iv) isopropoxide (Ti[OCH(CH_3_)_2_]_4_, Sigma Aldrich, 99.9+%), isopropanol, hydroiodic acid (HI, 57 wt% in H_2_O), hydrobromic acid (HI, 57 wt% in H_2_O), methylamine (CH_3_NH_2_, Aldrich, 33 wt% in absolute ethanol), aluminum, *N*,*N*-dimethylformamide (DMF, anhydrous, Sigma Aldrich), dimethylsulfoxide (DMSO, Anal. R. VWR chemicals, 99.5%), acetone, ethanol, Helmanex® detergent, chlorobenzene, 2-methoxyethanol (CH_3_OCH_2_CH_2_OH, Sigma Aldrich, 99.9%) and ethanolamine (H_2_NCH_2_CH_2_OH, Sigma Aldrich, 99%) were also used.

### b. Device fabrication

First, indium doped tin oxide (ITO) substrates were ultrasonically cleaned in acetone, detergent, deionized water and IPA, sequentially. The hole transporting layer (HTL), NiO_*x*_, was deposited at 4000 rpm for 30 s and annealed at 140 °C for 20 min. PEDOT:PSS (Clevios F HC) was spin-coated at 2500 rpm for 45 s and dried at 120 °C for 15 min, followed by IPA washing *via* spin-coating at 4000 rpm for 15 s and heating at 120 °C for 15 min. Then, HTL coated substrates were transferred into a glove box to deposit the perovskite film.

Mixed-cation–mixed-halide perovskite (Cs_0.05_(FA_0.83_MA_0.17_)_0.95_PbI_3–*x*_Br_*x*_) solution was prepared by mixing PbI_2_ (507.5 mg), FAI (172 mg), MABr (22.4 mg) and PbBr_2_ (73.5 mg) in 1 mL of dry *N*,*N*-dimethylformamide and dimethylsulfoxide solvent mixtures (with 4 : 1 (v/v) ratio), followed by stirring at 45 °C.^6^,^10^ Then, approximately 0.063 mol of CsI from 1.5 M stock solution (in DMSO) was added to the mixture and stirred overnight. The perovskite (PVS) solution was deposited on top of the HTL by a two-step spin-coating at 1500 rpm for 10 s with ramp 9 and at 6000 rpm for 30 s with ramp 2. During the second step, anti-solvent quenching was conducted *via* adding about 200 μL of chlorobenzene starting at the 23^rd^ s for about 3 s. Then, the film was annealed at 100 °C for 60 s. After the films had cooled, 2% (wt/wt) of PCBM in a mixture of chlorobenzene and chloroform (50 : 50 volume ratio) was spin-coated on top of the PVS film. Diluted TiO_*x*_ sol–gel solution was spin-coated on top of PCBM at 4000 rpm for 30 s, followed by annealing at 110 °C for about 5 min in ambient air. Finally, the inverted PSC fabrication was completed by thermal evaporation of 110 nm Al back electrode, which gave the PCBM/TiO_*x*_/Al sample and PCBM/Al control devices.

### c. Characterization

Surface morphologies of films were characterized by atomic force microscopy (AFM, Bruker Innova) and scanning electron microscopy (SEM, ZEISS 1540 XB cross-beam scanning microscope with a focused ion-beam (FIB) unit). Crystal structure, phase, and chemical information of the perovskite film were investigated by X-ray diffraction (Bruker D8 XRD system) employing Cu and Kα radiation source (*λ* = 1.5418 nm at 40 kV and 20 mA). Characteristic photocurrent density–photo voltage (*J*–*V*) response of the cells was recorded with a Keithley-2400-LV source meter with LabVIEW software. A LOT-QD solar simulator with 150 W xenon lamp emitting AM1.5 global spectrum and 100 mW cm^–2^ light intensity, which was calibrated using a standard Si reference diode, was used for irradiation. External-quantum efficiency (EQE) was measured using an optical setup consisting of a lock-in amplifier (SR830, Stanford Research Systems) and a Jaissle 1002 potentiostat functioning as a preamplifier. The devices were illuminated with light from a xenon lamp passing through a monochromator (Oriel Cornerstone). A filter wheel holding long-pass filters and a mechanical chopper was mounted between the xenon lamp and the monochromator. Chopping frequencies in the range of 10–200 Hz were used. A calibrated silicon diode (Hamamatsu S2281) was used as a reference for light intensity at each wavelength. A halogen lamp (Philips 50 W, 12 V) was used to provide a variable white light bias to the solar cells while EQE was measured.

Electrochemical impedance spectroscopy characterization was conducted under light perturbation in the frequency range of 1 MHz to 0.01 Hz using a Solaron potentiostat coupled with THORLABS DC2100 LED driver equipped with a detector (M590L3) and XM PhotoEchem software. Optical characterization was performed by recording photoluminescence decay, electroluminescence (EL) and photoluminescence (PL) measurements. To measure PL, the samples were excited with a VIOFLAME 405 nm laser (COHERENT UV GaN-based, 25 mW) and the signal was recorded with a Shamrock SR-303i monochromator and Andor™ iDus Si-CCD detector. EL characterization was performed using a Shamrock SR-303i monochromator and an Andor™ iDus Si-CCD detector to measure the signal and Keithley-2400-LV source meter to measure current under different voltage bias. Photoluminescence decay measurement was conducted using Shamrock (SR-303i–A) monochromator equipped with an intensified charge-coupled device camera [Andor iStar DH320T-18U-73 (gate step, 2.5 ns; gate width, 2.5 ns)] and Nd:YAG laser (Spit light Compact 100) emitting at 532 nm with a pulse length of ∼10 ns.

### d. Stability characterization

To test the relative stability of PSCs, maximum power point tracking of encapsulated solar cells was performed in ambient air as well as in a glove box with oxygen level in the range of 0.1–10 ppm under AM1.5 global spectrum illumination with continuous ventilation to keep the temperature low. *J*–*V* response of the devices was measured before and after maximum power point tracking. A white LED (XLamp CXA2011 1300K CCT) for ambient measurements and a 150 W xenon lamp for glove box measurements were used.

## Results and discussion

### a. Electronic and optical study

**Fig. 1:**
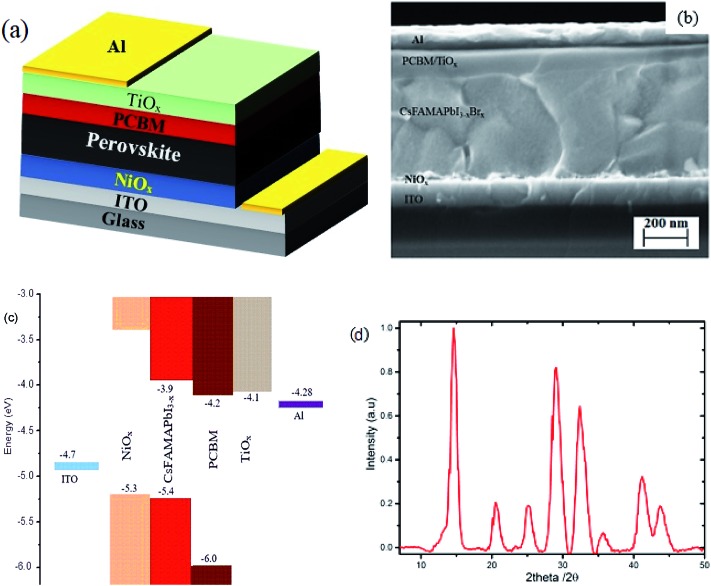
(a) Schematic of the overall structure, (b) cross-section SEM image of planar mixed-halide–mixed-cation PSC with TiO_*x*_ interlayer between PCBM and Al electrode, and (c) schematic energy band representation of the device. (d) Characteristic X-ray diffraction spectrum of mixed-halide–mixed-cation perovskite deposited on glass substrate.

Inverted mixed-halide–mixed-cation PSCs with TiO_*x*_ interlayer on top of the electron transporting layer (PCBM) were deposited on ITO coated glass substrates with low temperature processed NiO_*x*_ as the hole transporting layer, adopting the architecture ITO/NiO_*x*_/Cs_0.05_(FA_0.83_MA_0.17_)_0.95_PbI_3–*x*_Br_*x*_/PCBM/TiO_*x*_/Al, as shown in Fig. 1(a and b). Control devices were also prepared without TiO_*x*_ interlayer with ITO/NiO_*x*_/Cs_0.05_(FA_0.83_MA_0.17_)_0.95_PbI_3–*x*_Br_*x*_/PCBM/Al structure, as presented in Fig. S1(b).† X-ray diffractometry was applied to verify the crystal structure of the perovskite (Cs_0.05_(FA_0.83_MA_0.17_)_0.95_PbI_3–*x*_Br_*x*_) films. The films were deposited on oxygen plasma treated glass substrates following the same spin-coating parameters and heat treatment used for solar cell fabrication; the characteristic X-ray spectrum is shown in Fig. 1. The peak at 14.7° is a typical diffraction peak of (110) plane symmetry of tetragonal perovskite. Furthermore, the peaks at 20.6, 25.2, 29.0, 32.4, 35.7, 41.2 and 43.7° are the corresponding characteristic diffraction peaks of tetragonal perovskite with (112), (202), (220), (213), (311), (303) and (322) lattice planes, respectively, having lattice constants of 8.6 Å and 12.6 Å for *a* and *c*, respectively.^10^,^46^

**Fig. 2:**
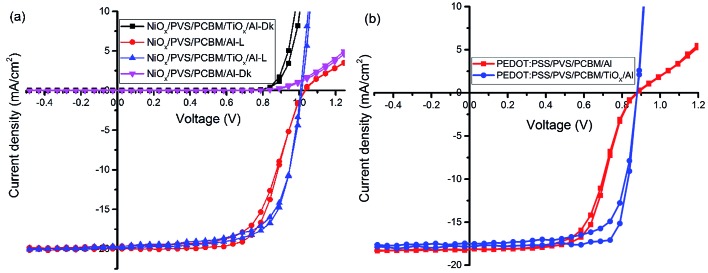
*J*–*V* characteristics of inverted mixed-cation–mixed-halide PSCs on (a) NiO_*x*_ HTL and (b) PEDOT:PSS HTL with TiO_*x*_ interlayer (PCBM/TiO_*x*_/Al interface) and control device (PCBM/Al structure) in dark (Dk) and under AM1.5 solar spectrum with 100 mW cm^–2^ light intensity illumination (*L*).

Fig. 2 shows the characteristic *J*–*V* response of mixed-halide–mixed-cation PSCs under 100 mW cm^–2^ illumination (AM1.5 global spectrum). The photovoltaic parameters are summarized in Table 1. *J*–*V* response of devices with PCBM/Al interface (devices without TiO_*x*_ interlayer) display less rectifying *J*–*V*, with the usual characteristic S-shape near open-circuit voltage (*V*_oc_), as shown in Fig. 2(a). This reduces the fill factor (FF = 65.3%) and hence power conversion efficiency (PCE = 13.5%). However, devices with TiO_*x*_ interlayer (PCBM/TiO_*x*_/Al interface) show rectifying *J*–*V* curves with much higher FF (69.4%) and improved PCE (∼14.6%) with substantially lower hysteresis, as shown in Fig. 2(a) and Table 1. Additionally, devices with TiO_*x*_ interlayer show lower characteristic resistance (*R*_s_ ∼ 16 Ω) compared to the devices without TiO_*x*_ interlayer (*ca.* 380 Ω), as shown in Table 1. The resistance element (*R*_s_) is related to serial resistance, which is calculated from the slope of the *I*–*V* curve by taking the last five points in the forward scan and first five points in the reverse scan. The other resistance value (*R*_p_), which is related to shunt resistance in the devices, was calculated from the slope of the *I*–*V* curve by taking the first five points in the forward scan and last five points in the reverse scan. Calculated *R*_s_ and *R*_p_ values are presented in Table 1.

**Table 1:** Summarized *J*–*V* characteristics (open-circuit voltage (*V*_oc_), short-circuit current (*J*_sc_), fill factor (FF), power conversion efficiency (PCE), parallel resistance (*R*_p_) and serial resistance (*R*_s_)) of PSCs with TiO_*x*_ interlayer (PCBM/TiO_*x*_/Al) and control (PCBM/Al) on NiOx and PEDOT:PSS (Clevios F HC) HTLs for forward (Fwd) and reverse scans (Rvs)

	*V*_oc_ (V)	*J*_sc_ (mA cm^–2^)	FF (%)	PCE (%)	*R*_p_ (Ω)	*R*_s_ (Ω)
**Devices with NiO**_***x***_ **as HTL**						
PCBM/Al – Rvs	1.03	20	65.6	13.5	14 493	385
PCBM/Al – Fwd	1.03	19.7	61.9	12.6	8509	373
PCBM/TiO_*x*_/Al – Fwd	1.04	20.1	66.2	13.9	5458	15
PCBM/TiO_*x*_/Al – Rvs	1.03	20.3	69.4	14.6	1788	16

**Devices with PEDOT:PSS as HTL**						
PCBM/TiO_*x*_/Al – Rvs	0.9	17.8	77.6	12.5	43 437	10
PCBM/TiO_*x*_/Al – Fwd	0.9	17.4	71	11	43 061	8
PCBM/Al – Fwd	0.9	18	58.7	9.2	1419	133
PCBM/Al – Rvs	0.9	18.1	61.2	9.7	9145	169

The same trend was observed for mixed-cation–mixed-halide PSCs based on PEDOT:PSS HTL, as shown in Fig. 2(b) and Table 1. Interestingly, devices with PCBM/TiO_*x*_/Al interface show improved FF of about 77.6% (reverse), PCE of ∼12.5% (reverse) and reduced *R*_s_ (10 Ω) compared to PCBM/Al interface based devices, which exhibit FF of about 61.2% (reverse), PCE of ∼9.7% (reverse) and about 12-fold higher *R*_s_ value of more than 130 Ω. This indicates that TiO_*x*_ reduces the charge extraction barrier between the electron transporting layer (PCBM) and Al electrode. The observed high series resistance and S-shaped *J*–*V* curve for devices without TiO_*x*_ interlayer might be correlated with corrosion of Al electrode *via* halide ion diffusion from the perovskite. As halide ions have small migration activation energy, these ions could diffuse through PCBM to react with Al; such a reaction causes formation of a thin insulating layer at the interface.^47^ External quantum efficiency and current density calculations for both devices (devices with and without TiO_*x*_ interlayer) show equivalent responses (Fig. S2†).

**Fig. 3:**
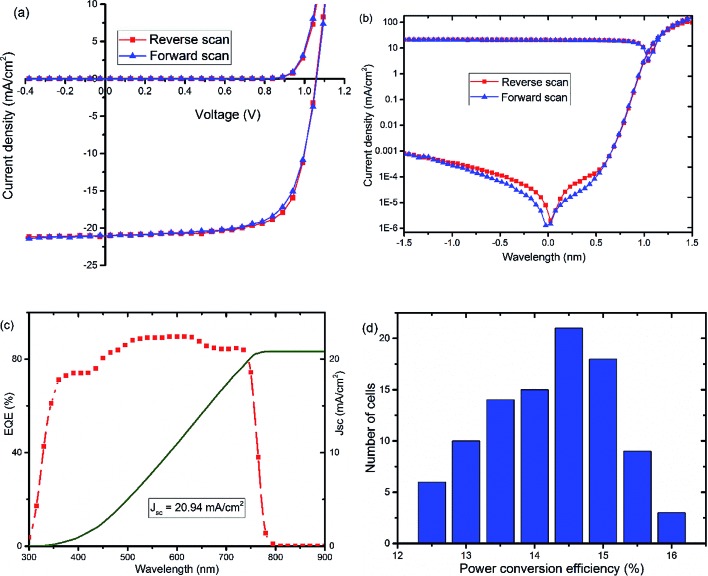
*J*–*V* curves, (a) linear and (b) semi-log plots, of optimized solar cells, glass/NiO_*x*_/Cs_0.05_(FA_0.83_MA_0.17_)_0.95_PbI_3–*x*_Br_*x*_/PCBM/TiO_*x*_/Al, recorded in dark (Dk) and under AM1.5 solar spectrum with 100 mW cm^–2^ light intensity illumination (*L*). (c) External quantum efficiency (EQE) spectrum and corresponding current density calculated from EQE data and (d) collective histogram of PCE distribution of PSCs with PCBM/TiO_*x*_/Al interface structure.

Further optimization of the processing conditions and thickness of the TiO_*x*_ interlayer yields a PSC device with negligible hysteresis and improved performance with *V*_oc_ of around 1.07 V, *J*_sc_ at 21.1 mA cm^–2^, FF of 72.5%, and PCE of 16%, as shown in Fig. 3(a and b) and S3.† The optimum thickness of TiO_*x*_, which gives the best performance, is about 10 nm deposited on top of an approximately 80 nm thick PCBM layer. The characteristic photovoltaic parameters of the best performing device are summarized in Table 2. EQE of the best device improved to 85% with integrated current density of 20.94 mA cm^–2^, as displayed in Fig. 3(c). The histogram for the average PCE values of devices is shown in Fig. 3(d). As indicated in the figure, the highest PCE is above 16%, with average device performance in the range of 14.5–15% with good reproducibility.

**Table 2:** Characteristic *J*–*V* parameters (open-circuit voltage (*V*_oc_), short-circuit current (*J*_sc_), fill factor (FF), power conversion efficiency (PCE), parallel resistance (*R*_p_) and serial resistance (*R*_s_)) of optimized mixed-cation–mixed-halide PSCs with TiO_*x*_ interfacing (PCBM/TiO_*x*_/Al) in forward (Fwd) and reverse scans (Rvs) under 100 mW cm^–2^ light intensity illumination

	*V*_oc_ [V]	*J*_sc_ [mA cm^–2^]	FF [%]	PCE [%]
R-scan	1.07	21.1	72.4 ± 0.6	16.3 ± 0.2
F-scan	1.07	21	70.7 ± 0.7	15.9 ± 0.2

**Fig. 4:**
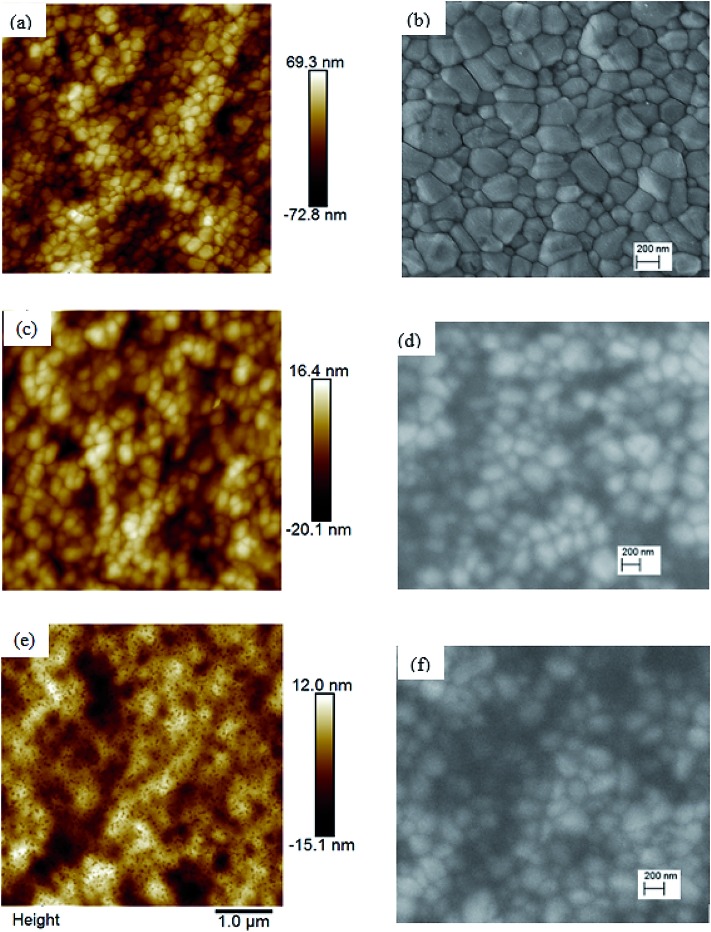
Atomic force microscopy (AFM, left) and scanning electron microscopy (SEM, right) images of (a and b) mixed-cation–mixed-halide perovskite films, (c and d) mixed-cation–mixed-halide perovskite with PCBM on top, and (e and f) mixed-cation–mixed-halide perovskite/PCBM with TiO_*x*_ on top. Films are deposited on ITO substrate covered with NiO_*x*_ particles.

To investigate the reasons leading to improved photovoltaic performance of PSCs with TiO_*x*_ interlayer, surface characterization of TiO_*x*_ interlayered perovskite (PVS)/PCBM/TiO_*x*_ structure, PVS films and PVS films covered with PCBM was conducted using atomic force microscopy (AFM) and scanning electron microscopy (SEM). The surface morphology of mixed-cation–mixed-halide perovskite film deposited on NiO_*x*_, as shown in Fig. 4(a) and (b), is characterized by well packed, dense and pinhole-free film with grain sizes in the range of 50–500 nm. The grain size in the AFM image is consistent with the SEM results. The root-mean-square (RMS) roughness calculation from AFM data shows the grain size of 21.9 nm. Deposition of about 80 nm PCBM on top of the perovskite layer results in a significantly smoother surface, as shown in Fig. 4(c) and (d), with average RMS roughness of 6.9 nm. This shows the effective coverage of perovskite film with PCBM, which is an essential requirement to avoid direct contact of the perovskite with the top electrode and surrounding ambient air, as it is highly sensitive to polar solvents. With the deposition of about 15 nm TiO_*x*_ layer on top of PCBM, a smooth film with sparsely distributed nanodot features on the surface is formed, as shown in Fig. 4(e) and (f). The presence of TiO_*x*_ layer further decreases the RMS roughness to 4.6 nm. This indicates that the deposition of TiO_*x*_ layer improves surface coverage of the ETL. This could heal the defects of PCBM film and reduce the probability of direct contact between the photoactive layer and Al back electrode. Preliminary Kelvin-probe microscopic (KPFM) experiments also indicate the role of TiO_*x*_ in decreasing the charge trapping and improving the charge transport through the interface.^48^ Moreover, such a morphological change can modify the interfacial contact area, which will have a significant effect on the charge extraction process across the interface.

**Fig. 5:**
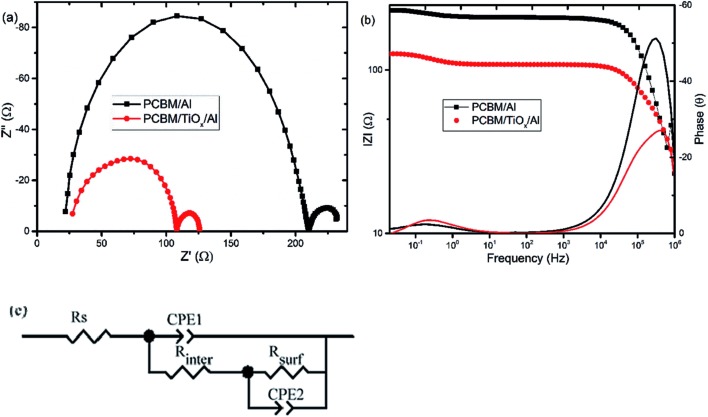
(a) Nyquist and (b) Bode plots of characteristic electrochemical impedance spectroscopy (EIS) responses for PSCs with TiO_*x*_ interlayer (PCBM/TiO_*x*_/Al) and control device (PCBM/Al) scanned in the frequency range of 1 MHz to 20 mHz and 10% light modulation under 8 mW cm^–2^ LED light intensity. (c) Equivalent circuit model for solar cells.

Electrochemical impedance spectroscopy (EIS) and intensity modulated photovoltage spectroscopy (IMVS) response of PSCs with PCBM/TiO_*x*_/Al and PCBM/Al n-contact structure were measured to further investigate the effect of TiO_*x*_ interlayer on electron dynamics across the interface. EIS response of PSCs was measured over the frequency range from 1 MHz to 0.02 Hz under 8 mW cm^–2^ LED light intensity perturbation. Nyquist plots of EIS response are shown in Fig. 5(a) and corresponding Bode plots of EIS response for both devices are shown in Fig. 5(b). EIS response for both devices shows two characteristic peaks: the one at higher frequency (1 MHz to 10 kHz) is associated with charge carrier transport resistance (*R*_inter_) and the peak lower frequency (10 Hz to 20 mHz) is attributed to impedance of trap states (charge recombination) within the perovskite film and at the interface of charge transport layers.^5^,^35^,^49^ The equivalent circuit model for the solar cells is shown in Fig. 5(c) and fitting parameters for the equivalent circuit and EIS response of devices are shown in Table S1.† Control device (PCBM/Al structure) and devices with TiO_*x*_ interlayer (PCBM/TiO_*x*_/Al) show characteristic high frequency EIS resistance, corresponding to charge flow resistance (*R*_inter_) of 189 and 38.5 Ω, respectively. In low frequency EIS response, control devices show higher characteristic resistance (23 Ω, Table S1†) compared to devices with TiO_*x*_ interlayer (16 Ω, Table S1†). This might be associated with the presence of more trap states^50^ for devices without TiO_*x*_ interlayers, which is consistent with the preliminary KPFM observations.

**Fig. 6:**
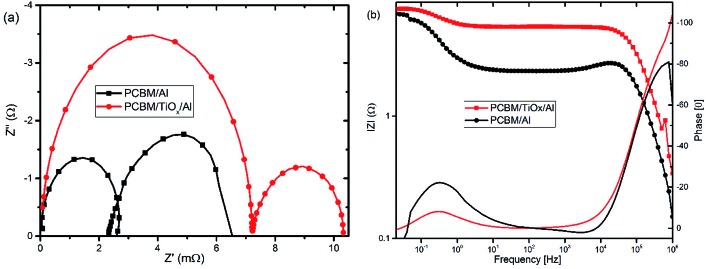
Characteristic (a) Nyquist and (b) Bode plots of intensity modulated photovoltage spectroscopy (IMVS) responses for PSCs with TiO_*x*_ interlayer (PCBM/TiO_*x*_/Al) and control device (PCBM/Al) scanned in the frequency range of 1 MHz to 20 mHz and 10% light modulation under 8 mW cm^–2^ LED light intensity.

In parallel with the abovementioned results, high frequency IMVS response in Nyquist plots presented in Fig. 6(a) and corresponding Bode plots shown in Fig. 6(b) are associated with recombination resistance (*R*_rec_) of the devices.^50^ Devices with TiO_*x*_ interlayer show higher recombination resistance features relative to devices without TiO_*x*_ interlayer, which is analogous to the EIS response. This indicates the improvement in charge carrier extraction energetics for devices with TiO_*x*_ interlayer, which is in agreement with the observed lower *R*_s_ values in the *J*–*V* response (Table 1).

**Fig. 7:**
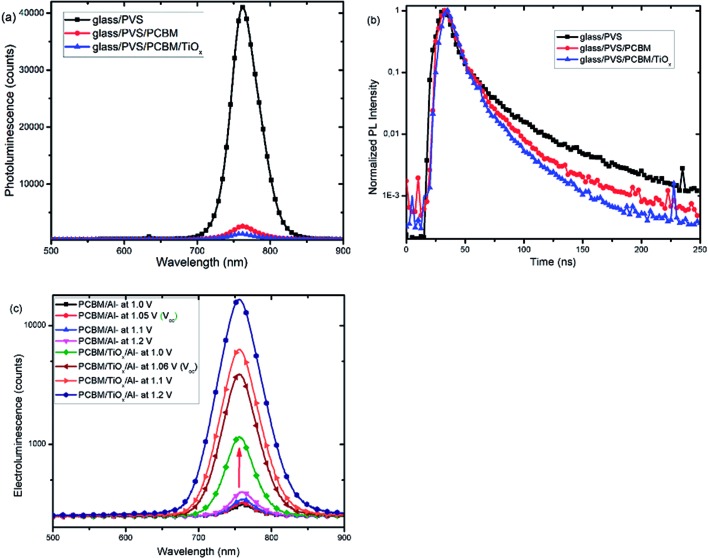
(a) Photoluminescence (PL) spectra and (b) photoluminescence decay spectra of mixed-cation–mixed-halide perovskite films (PVS) and PVS covered with PCBM (PVS/PCBM) and with additional TiO_*x*_ top layer (PVS/PCBM/TiO_*x*_). (c) Electroluminescence (EL) spectra as a function of voltage bias of p–i–n based mixed-cation–mixed-halide perovskite SCs with TiO_*x*_ interlayer (PCBM/TiO_*x*_/Al interfacing) and control device (PCBM/Al interfacing) under laser excitation (405 nm).

Furthermore, photoluminescence (PL) characterization of perovskite film, perovskite films covered with PCBM (PVS/PCBM), and PCBM coated with TiO_*x*_ (PVS/PCBM/TiO_*x*_) on glass substrate was conducted, as shown in Fig. 7(a). Devices with TiO_*x*_ interlayer show stronger quenching compared to devices without TiO_*x*_ interlayers. Similarly, photoluminescence decay spectra, as shown in Fig. 7(b), also display faster PL decay in devices with TiO_*x*_ interlayer, relative to devices without TiO_*x*_ interlayer. This indicates the decrease in charge trapping sites at the ETL interface for films with TiO_*x*_ interlayer.

Fig. 7(c) presents the electroluminescence (EL) spectra of PSCs with TiO_*x*_ interlayer (PCBM/TiO_*x*_/Al) and control devices (PCBM/Al). Devices with TiO_*x*_ interlayer show stronger EL response at a given bias compared to the control devices, which indicates the improvement in charge injection. This could be related to the reduction of charge trapping and recombination conduits near the ETL and back electrode interfaces and reduction in charge injection barrier between the ETL and Al electrode due to TiO_*x*_ interlayer.

### b. Stability study

**Fig. 8:**
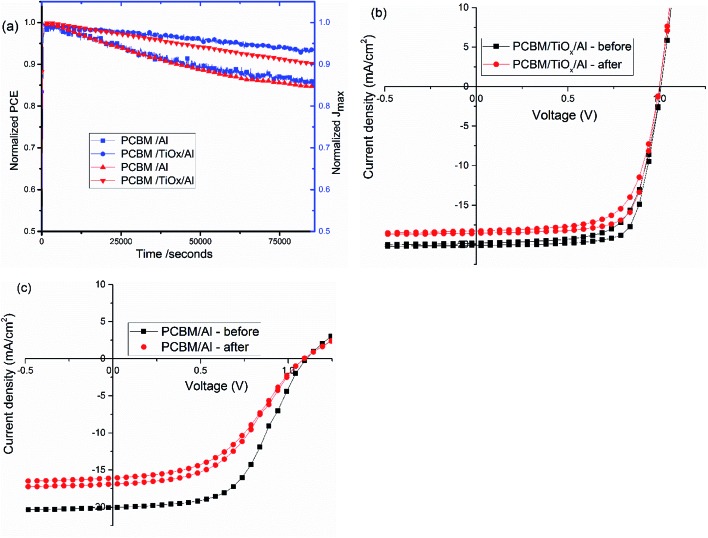
(a) Steady state power conversion efficiency (PCE) and maximum current density (*J*_max_) under continuous illumination for about 22 h in ambient environment; characteristic *J*–*V* curves before and after continuous maximum power point tracking for PSCs with (b) PCBM/TiO_*x*_/Al and (c) PCBM/Al interfacing under 100 mW cm^–2^ light intensity illumination.

Stability is a key issue in PSCs as the photoactive material is prone to moisture, oxygen, UV light and temperature degradation.^32^–^34^,^51^ Herein, stability was monitored for mixed-halide–mixed-cation PSCs with and without TiO_*x*_ interlayer between PCBM and Al electrode. Maximum power point tracking measurements were conducted in ambient air under AM1.5 solar spectrum with 100 mW cm^–2^ intensity illumination. As shown in Fig. 8, devices with PCBM/TiO_*x*_/Al interfacing are more stable than devices without TiO_*x*_ interlayer (PCBM/Al). Devices with TiO_*x*_ interlayer show only about 7% and 10% loss of *J*_max_ and PCE, respectively, after 22 h continuous operation, while cells with PCBM/Al ETL interface show about 14% and 15% decrease of *J*_max_ and PCE, respectively, as shown in Fig. 8(a). The characteristic *J*–*V* response of solar cells was also measured before and immediately after the power tracking experiment. As presented in Fig. 8(b and c) and Table 3, both devices show a decrease in *J*_sc_, FF and PCE. However, for devices with PCBM/Al interfacing, the resistance element (*R*_s_) under illumination increases from 253 to 372 Ω after 22 h continuous power tracking in ambient air. For PCBM/TiO_*x*_/Al interfaced devices, the *R*_s_ value is almost the same after operation. This further indicates the stabilizing role of TiO_*x*_ interlayer in ambient air.

**Table 3:** Average *J*–*V* characteristics of devices with TiO_*x*_ interlayer (PCBM/TiO_*x*_/Al) and control (PCBM/Al) measured before maximum power tracking and after tracking for 22 h under 1 sun (100 mW cm^–2^)

ETL Interface	*V*_oc_ [V]	*J*_sc_ [mA cm^–2^]	FF [%]	PCE [%]	*R*_p_ [Ω]	*R*_s_ [Ω]	Measured
PCBM/TiO_*x*_/Al	1.01	23.9	70.1	16.9	10 151	21	Before
PCBM/TiO_*x*_/Al	1	22.1	69	15.2	53 588	20	After
PCBM/Al	1.09	20.5	53.3	11.9	19 819	284	Before
PCBM/Al	1.09	16.5	46.1	8.3	9049	366	After

**Fig. 9:**
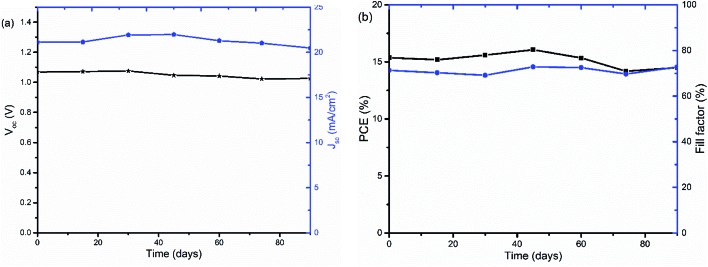
Stability of PSCs with ITO/NiO_*x*_/Cs_0.05_(FA_0.83_MA_0.17_)_0.95_PbI_3–*x*_Br_*x*_/PCBM/TiO_*x*_/Al device structure under long term storage in a glove box: (a) open-circuit-voltage *V*_oc_ (black) and short-circuit-current *J*_sc_ (blue) and (b) power conversion efficiency PCE (black) and fill factor (FF) (blue) as a function of storage time.

The stability of PSCs with TiO_*x*_ interlayers was also studied under long term storage in a glove box under oxygen and water level in the range of 0.1 to 10 ppm. The solar cells were stored for more than 90 days and their *J*–*V* curves were measured regularly. As shown in Fig. 9, the device was quite stable with about 4.7% *V*_oc_ loss and 5% PCE decrease upon aging.

The rapid degradation of PCBM/Al structure PSCs (Fig. 8(a and c)) could be related to insufficient protection, leading to liberation of MAI from the PVS to the PCBM/Al interface, rapid chemical reaction between the Al electrode and the perovskite, and/or further exposure to the ambient environment.^52^,^53^ Another reason could be the degradation of the PCBM layer itself through adsorption of oxygen and water.^54^ As shown in the AFM image (Fig. 4(c)), RMS roughness decreases when TiO_*x*_ interlayer is deposited on top of PCBM, which could indicate the improvement in surface smoothness and surface coverage. TiO_*x*_ could heal pore sites and surface defects on PCBM, which might block direct infiltration of Al electrode to the perovskite layer and ion flow to the Al electrode. This prevents corrosion of the back contact electrode due to migrating mobile halide ions from the perovskite layer.^47^ Additionally, TiO_*x*_ interlayer could protect the PCBM from ambient air and improve stability. Overall, deposition of TiO_*x*_ interlayer between PCBM and Al electrode plays a dual role of reducing the energy barrier for carrier extraction and improving the stability of the solar cells.

## Conclusion

In summary, we demonstrated the improvement in photovoltaic parameters and stability of inverted mixed-cation–mixed-halide perovskite solar cells *via* interfacing the PCBM and Al with low temperature sol–gel processed TiO_*x*_. Devices with ETL interfacing using PCBM/TiO_*x*_ display lower resistance values (in the range of 5–30 Ω) relative to control devices, resulting in improved rectification in characteristic *J*–*V* curves and improved FF and PCE values. Microscopic observation of surface morphology illustrates the decrease in surface roughness. EIS and IMVS measurements show high charge transfer impedance for devices without TiO_*x*_ interlayer and higher recombination impedance for devices with TiO_*x*_ interlayer. PL and EL characterization indicate the improvement in charge carrier extraction process for devices with PCBM/TiO_*x*_/Al relative to that for the control devices (PCBM/Al). Moreover, devices with TiO_*x*_ interlayer show better stability under continuous operation in ambient air and long term storage in the glove box. Therefore, based on our results, we can conclude that TiO_*x*_ interfacing improves device performance and stability.

## Conflicts of interest

We have no conflicts of interest to disclose.

## Supplementary Material

Supplementary information
