# Supplementary material for: Inverted (p–i–n) perovskite solar cells using a low temperature processed TiOx interlayer
Source: RSC Adv. 2018 Jul 10;8(44):24836–46. doi: 10.1039/c8ra03993c (PMC6333247; doi:10.1039/c8ra03993c)
Supplement: RA-008-C8RA03993C-s001 [file RA-008-C8RA03993C-s001.pdf]

Electronic Supplementary Material (ESI) for RSC Advances.
This journal is © The Royal Society of Chemistry 2018

Electronic Supplementary Information

Inverted (p-i-n) Perovskite Solar Cells Using Low Temperature Processed TiO_x Interlayer

Bekele Hailegnaw,*[a] Getachew Adam,[a,b] Herwig Heilbrunner,[a] Dogukan H. Apaydin,[a] Christoph Ulbricht,[a] Niyazi Serdar Sariciftci,[a] Markus C. Scharber*[a]

[a] *Linz Institute for Organic Solar Cells (LIOS), Institute of Physical Chemistry, Johannes Kepler University Linz, Altenbergerstrasse 69, 4040 Linz, Austria.*

[b] *Department of Industrial Chemistry, College of Applied Science, Addis Ababa Science and Technology University (AASTU), P.O.Box 16417, Addis Ababa, Ethiopia*

Corresponding author: Markus Clark Scharber
Email: markus_clark.scharber@jku.at

Experimental Details

a. NiO_x Film Preparation

Nickel oxide was synthesized following the procedure described by Young, et al. [1] About 2 % (wt/wt) of NiO_x powder was mixed with deionized water (DIW) (20 mg per 1 mL of DIW), and treated with ultrasound sonicator (UP50H, 50 Watt, frequency 30 kHz) for five to six round with each round for about five minutes to further crash the particles. In between each round the mixture was kept in an ultra-sonication bath to avoid aggregation of particles. The solution was then filtered with 0.45 µm pore size filter and spin-coated on ITO substrates.

b. Synthesis of Organic Halides

To synthesize methyl ammonium iodide (MAI), methylamine was added in a round-bottom flask and immersed into an ice bath to keep the reaction temperature at 0 °C and equimolar amount of hydroiodic acid was added drop-by-drop with continuous stirring. The reaction mixture was left under stirring in the ice bath for 2 h. The solvent was then evaporated using a Rota vapor (BUCHI Rotavapor R-114 coupled with a BUCHI water bath B-480). The resulting crystals were re-dissolved in a small amount of ethanol and precipitated by adding diethyl ether and decanted. Washing with diethyl ether was repeated until clean white crystals were obtained. The crystals

were then filtered with a Buchner funnel under vacuum and transferred to a round bottom flask followed by freeze drying. Finally the dried, white powder of MAI was transferred into a vial and kept in a nitrogen glove box. Formamidine acetate salt (99 %) and Hydroiodic acid (HI, 57 wt % in H_2O) in 1:1 mol ratio was used to synthesize formamidinium iodide ($CH(NH_2)_2I$) following the same synthesis procedure used for MAI. The same procedure was applied to synthesize methyl ammonium bromide (CH_3NH_3Br) using methyl amine and hydrobromic acid (HBr, $\geq$ 48 wt % in H_2O) as a precursor materials.

c. Synthesis TiO_x Solution

The sol-gel synthesis TiO_x precursor solution was based on the procedure reported by S. H. Park et al.[2] A three-necked flask (100 mL) was used to mix the precursor material. It was first dried at about 120 °C with flowing dry nitrogen (N_2) to remove any moisture. Then, a 2.5 mL of titanium (IV) isopropoxide ($Ti[OCH(CH_3)_2]_4$), 10 mL of 2-methoxyethanol ($CH_3OCH_2CH_2OH$) and 1 mL of ethanolamine ($H_2NCH_2CH_2OH$) were sequentially injected into the three-necked flask at room-temperature (RT).

The precursor solution was stirred for 60 min at room temperature under N_2 atmosphere and then heated for 60 min at 80 °C (using a silicon oil bath), followed by heating at 120 °C for 60 min with continuous stirring to distilled off low boiling solvent. The solution was then transformed into a low-density gel with dark-wine color. The mixture was cooled to room temperature and isopropyl alcohol (IPA, 5 mL) was added to prepare TiO_x sol-gel precursor solution. The TiO_x sol-gel precursor was further diluted in IPA with 1:150 (v/v) ratio to be used in the device.

Result

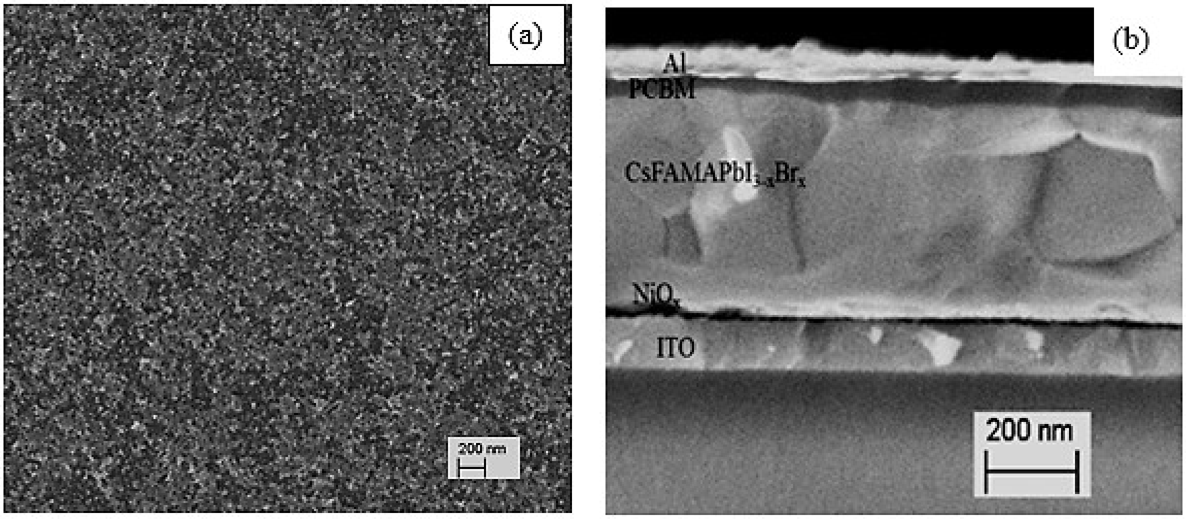

Figure S1. (a) Topographic SEM image of NiO_x nanoparticles on ITO-coated glass substrate, and (b) cross-section SEM image of mixed-halide mixed-cation PSCs without TiO_x interlayer (PCBM/Al structure).

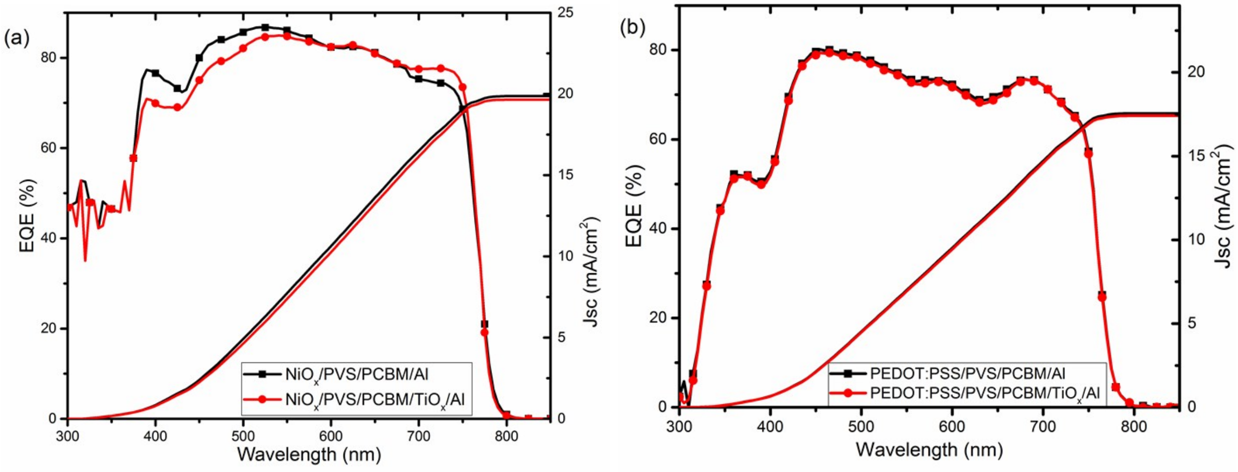

Figure S2. External quantum efficiency (EQE) of inverted PSCs with, (a) NiO_x HTL, and (b) PEDOT:PSS HTL with and without TiO_x interlayer on top of PCBM and the corresponding current density calculated from the EQE data.

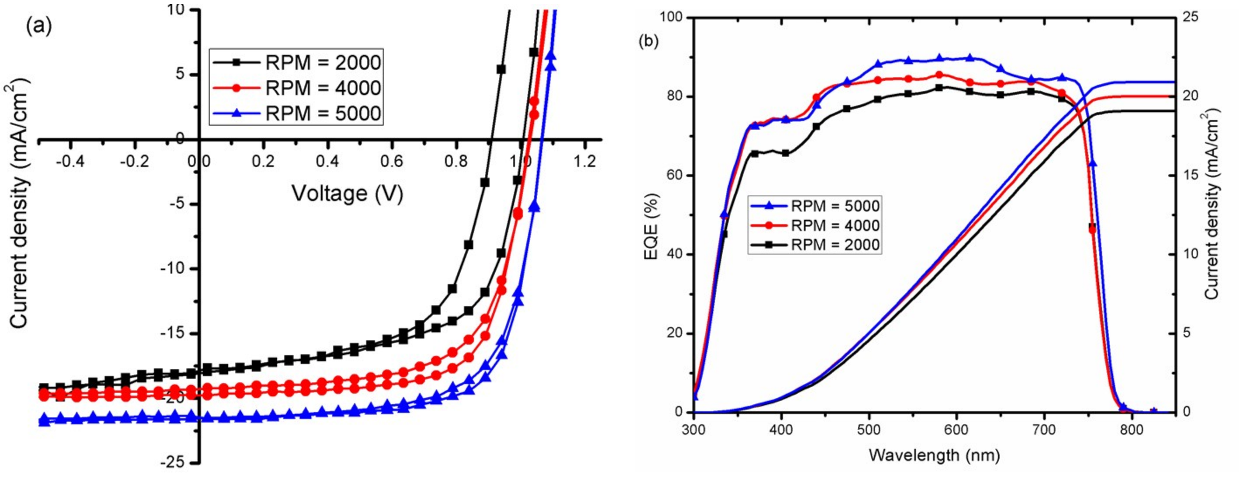

Figure S3. (a) J-V curves, and (b) external quantum efficiency (EQE) as well as the calculated current density from the EQE data of PSCs with different thickness of TiO_x interlayer processed at various spin-coating speeds (i.e. 2000, 4000 and 5000 revolutions-per-minute (RPM) yielding TiO_x films thickness of about 35 to 40, 15 to 20 and 10 to 15 nm, respectively).

Table S1. Fitting parameters of the equivalent circuits for the characteristic EIS response of PSCs with TiO_x interlayer (PCBM/TiO_x/Al) and control devices (PCBM/Al).

**Device structure**	**Rs (Ω)**	**$R_{internal}$ (Ω)**	**CPE1**		**$R_{surface}$ (Ω)**	**CPE2**	
			**T1**	**P1**		**T2**	**P2**
PCBM/AL	20	189	2.5×10^{7}	0.925	23	0.04	0.85
PCBM/TiO_x/Al	22	38.5	2.0×10^{7}	0.9	16	0.06	0.85

Reference

1 X. Yin, P. Chen, M. Que, Y. Xing, W. Que, Ch. Niu, J. Shao, *ACS Nano,* 2016, 10, 3630-3636.

2 S. H. Park, A. Roy, S. Beaupre, S. Cho, N. Coates, J. S. Moon, D. Moses, M. Leclerc, K. Lee, A. J. Heeger, *Nat. Photonics*, 2009, 3, 297-303.
